# Direct observation of pitting corrosion evolutions on carbon steel surfaces at the nano-to-micro- scales

**DOI:** 10.1038/s41598-018-26340-5

**Published:** 2018-05-22

**Authors:** Peng Guo, Erika Callagon La Plante, Bu Wang, Xin Chen, Magdalena Balonis, Mathieu Bauchy, Gaurav Sant

**Affiliations:** 1 Laboratory for the Chemistry of Construction Materials (LC2), Department of Civil and Environmental Engineering, University of California, Los Angeles, CA 90095 United States; 2 Department of Materials Science and Engineering, University of California, Los Angeles, CA 90095 United States; 3 Department of Bioengineering, University of California, Los Angeles, CA 90095 United States; 4 Laboratory for the Physics of Amorphous and Inorganic Solids (PARISlab), Department of Civil and Environmental Engineering, University of California, Los Angeles, CA 90095 United States; 5 California Nanosystems Institute, University of California, Los Angeles, CA 90095 United States

## Abstract

The Cl^−^-induced corrosion of metals and alloys is of relevance to a wide range of engineered materials, structures, and systems. Because of the challenges in studying pitting corrosion in a quantitative and statistically significant manner, its kinetics remain poorly understood. Herein, by direct, nano- to micro-scale observations using vertical scanning interferometry (VSI), we examine the temporal evolution of pitting corrosion on AISI 1045 carbon steel over large surface areas in Cl^−^-free, and Cl^−^-enriched solutions. Special focus is paid to examine the nucleation and growth of pits, and the associated formation of roughened regions on steel surfaces. By statistical analysis of hundreds of individual pits, three stages of pitting corrosion, namely, induction, propagation, and saturation, are quantitatively distinguished. By quantifying the kinetics of these processes, we contextualize our current understanding of electrochemical corrosion within a framework that considers spatial dynamics and morphology evolutions. In the presence of Cl^−^ ions, corrosion is highly accelerated due to multiple autocatalytic factors including destabilization of protective surface oxide films and preservation of aggressive microenvironments within the pits, both of which promote continued pit nucleation and growth. These findings offer new insights into predicting and modeling steel corrosion processes in mid-pH aqueous environments.

## Introduction

Pitting corrosion is a damaging form of localized metallic corrosion that has been a subject of research for several decades^1,2^. Although the formation of pits consumes only a relatively small amount (mass) of material, the role of pits in serving as defect sites for crack initiation and continuing corrosion can be significant^3,4^. Furthermore, halide ions such as chloride (Cl^−^) are well-known to accelerate the pitting corrosion of alloys such as steel^5–7^. Numerous mechanisms have been proposed to explain pitting initiation in the presence of Cl^−^ which involves the breakdown of a surficial oxidation/passivation film by processes including: penetration of Cl^−^ species through the film, and ion-adsorption and local thinning, which ultimately leads to film breakdown^2,8^.

But, the initiation and progression of pitting corrosion is difficult to study quantitatively for a number of reasons. First, the small size of pits makes their detection challenging especially at early stages of pitting when pits feature diameters ≤20 µm and depths of only a few tens of nanometers^9–11^. Second, pits may be obscured by corrosion products that may form (concurrently with pits) on metallic surfaces^11–13^. While electrochemical studies may be applied to examine pitting – these approaches only assess bulk (“average”) behavior – without being able to resolve microscale features^2,14–16^. More recently, electrochemical scanning tunneling microscopy (ECSTM) has been used to study oxide layer removal and pitting initiation on metal surfaces^17,18^, whereas atomic force microscopy (AFM)^19^ has been utilized to observe the morphology of single pits. These techniques have provided valuable insights into pit growth without invoking any assumptions regarding the active area of pitting sites, or regarding the current densities of single pits^8^. However, ECSTM can only probe a small field of view (FOV) on the order of a few square microns, whereas AFM is unable to image sharp and deep pits (>10 µm) due to its limited vertical range. More recently, an electrochemical surface forces apparatus (EC-SFA) has been used to resolve corrosion pits in confined spaces *in situ*, and in the presence of Cl^−^ ions and applied potential, albeit with restrictions in the nature of geometries that can be examined^20^.

To overcome these limitations, vertical scanning interferometry (VSI) is used for the first time to study the initiation and development of pitting corrosion. In brief, VSI uses the interference of light having short coherence lengths to assess surface topography. As such, a vertical range of up to a few millimeters that can be sampled at up to sub-nanometer vertical resolution, and up to a sub-micron lateral resolution, are achieved. This allows for the detection of pits from their nucleation until maturity. Furthermore, to acquire statistically valid data, multiple images can be tiled to generate composite images that represent sample areas of up to tens of square millimeters. These capabilities enable observations of pit nucleation and propagation, at unparalleled resolution over representative surface areas encompassing 1000 s of individual pits. As such, this study unambiguously elucidates new insights into the kinetics and mechanisms of pitting corrosion in (medium) carbon steels. Although the electrochemical properties of this material in the presence of diverse reaction solutions have been extensively studied^21–23^, the topography evolution of their surfaces has been much less monitored and quantitatively analyzed. To demonstrate this new approach of investigating pitting corrosion, an AISI 1045 steel was exposed to deionized (DI) water and Cl^−^ containing solutions to gain quantitative insights into surface morphology evolutions as corrosion proceeds and to clarify the effects of aqueous Cl^−^ species on amplifying corrosion activity.

## Results and Discussion

### Heterogeneous corrosion of AISI 1045 steel surfaces

**Figure 1:**
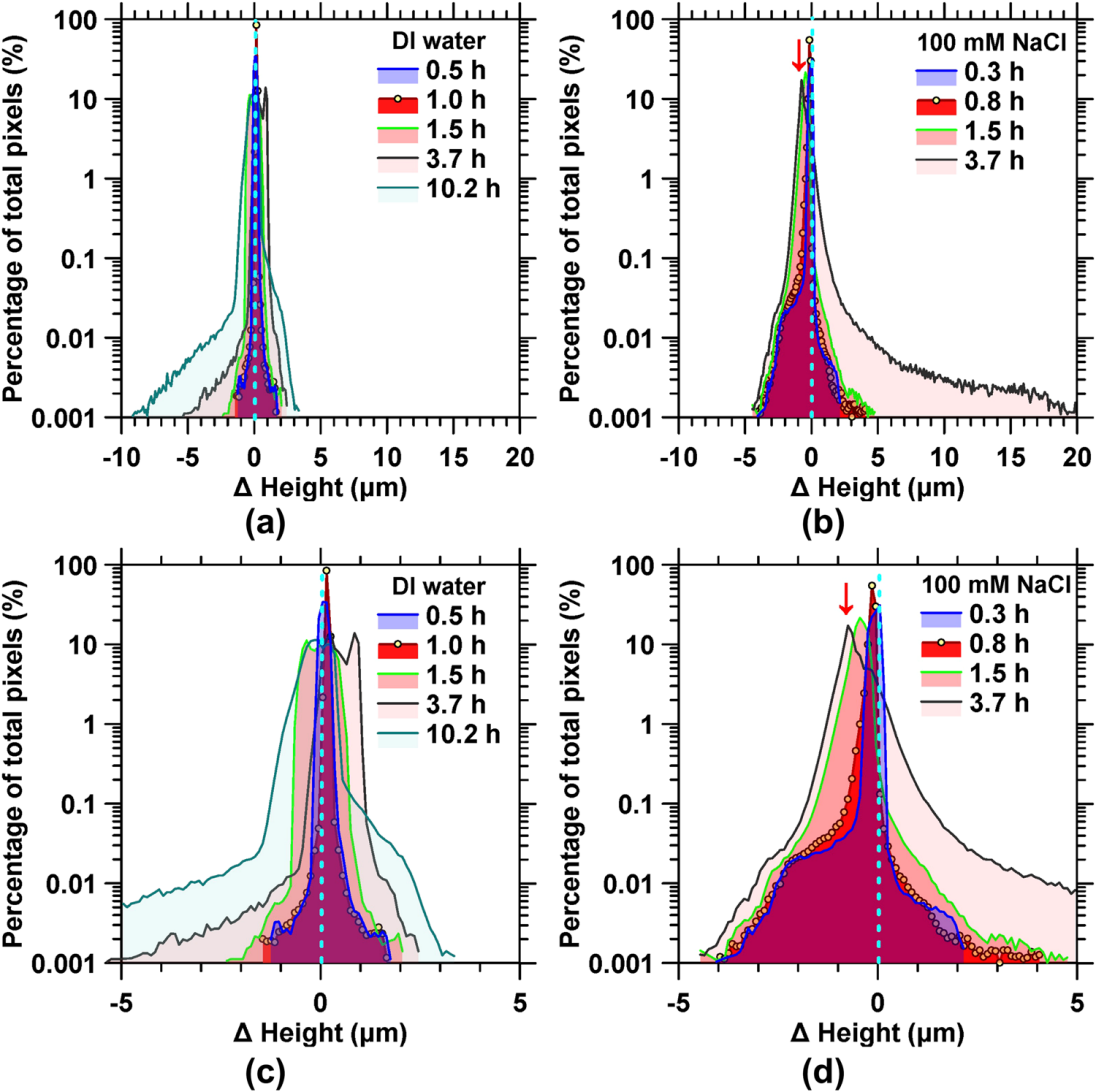
Representative frequency diagrams showing the distribution of height differences across different pixel locations for AISI 1045 steel reacting with: **(a)** DI water, and **(b)** 100 mM NaCl solution. The height difference is calculated by subtracting the heights at a given *(x*, *y)* location at the respective times indicated in the legend from that at *t* = 0 h. As such, negative height differences indicate mass loss (e.g., pit formation, and surface roughening), and positive height differences indicate the deposition of corrosion products. **(c)** and **(d)** are the distributions over the height range of −5 to 5 µm, showing in more detail the respective height distributions in **(a)** and **(b)**.

Careful examination of 3D topography data acquired using VSI reveals the highly non-uniform, i.e., heterogeneous, progress of corrosion in both deionized (DI) water and 100 mM NaCl solutions. Surface degradation proceeded by the nucleation, accumulation and growth of corrosion pits. To quantitatively examine the heterogeneity of corrosion, the height difference of reacting surfaces was calculated pixel-by-pixel relative to the unreacted *t*_0_ surface, across each time-spaced FOV. The frequency distribution of height differences was then monitored over time as shown in Fig. 1. It should be noted that negative and positive height differences indicate either the net removal from, or the net deposition of material on to the surface, respectively. As corrosion proceeds, distinct changes in these distributions become evident. Expectedly, at *t*_0_, the distribution was characterized by a sharp peak centered at 0 µm. This ‘peak’ persisted even at later times indicating that the majority of the surface featured a largely uniform, unchanging topography across the FOV, and that only localized domains on the surface (i.e., encompassing less than 1% of all pixels) showed substantial changes that are characteristic of pit formation^8^.

The surface height evolutions in DI water and in 100 mM NaCl solutions are distinct, as indicated in Fig. 1. In DI water, corrosion is associated primarily with the deepening of localized pits, as suggested by the emergence of the ‘low-frequency tail’ shown in Fig. 1(a). The largest surface retreat measured, which affected approximately 0.001% of all pixels, or equivalently 0.001% of the surface area, was about 10 µm after around 10.2 h of reaction (Fig. 1a). In the presence of 100 mM NaCl on the other hand, the widespread accumulation of localized pits that are up to 5 µm deep resulted in the development of a secondary peak centered at Δ*h* ≈ −2 µm after only 0.33 h of solution contact. In time, this secondary peak became indistinguishable from the initial peak, suggesting that in general, rapid pit nucleation and growth occurred until the entire steel surface was extensively pitted, as illustrated below. The red arrows in (Fig. 1b,d) indicate a shift in the position of the main peak, suggesting that corrosion resulted in an overall surface retreat on the order of 0.7 µm after around 3.7 h in the presence of NaCl. In turn, the average surface height decrease that can be related to the amount of mass loss is substantially higher in the presence of aqueous Cl^−^ than in DI water.

The corrosion process is accompanied by the formation of corrosion products. In DI water, simultaneous to pitting, a broadly homogeneous height increase of about 3 µm was observed over around 0.1% of the imaging area, indicating the formation and growth of a surface layer of corrosion products. Analyses carried out using µ-Raman spectroscopy and energy dispersive x-ray spectroscopy (SEM-EDS) have identified these corrosion products as consisting of lepidocrocite (γ-FeOOH), goethite (α-FeOOH), and the iron oxides hematite (Fe_2_O_3_) and magnetite (Fe_3_O_4_)^24,25^. On the other hand, up on exposure to 100 mM NaCl, the frequency distribution of the surface height change features a long tail signifying a height increase of up to >20 µm after 3.7 h of solution exposure (Fig. 1b). This indicates substantial formation of corrosion products on AISI 1045 steel reacting with Cl^−^-containing solutions, consistent with previous observations^26^. Indeed, compositional data acquired using µ-Raman spectroscopy indicates the presence of lepidocrocite and akageneite (β-FeOOH) on AISI 1045 steel exposed to both 10 mM and 100 mM NaCl following 24 hours of solution contact (see Fig. S1), consistent with previous reports^27–29^. It should be noted that the observed height increases in the presence of Cl^−^ are also localized, with only a small fraction of the surface (0.02% area percentage) showing an increase of 10 to 20 µm. The specific surficial features that are associated with these frequency distributions are further discussed below.

**Figure 2:**
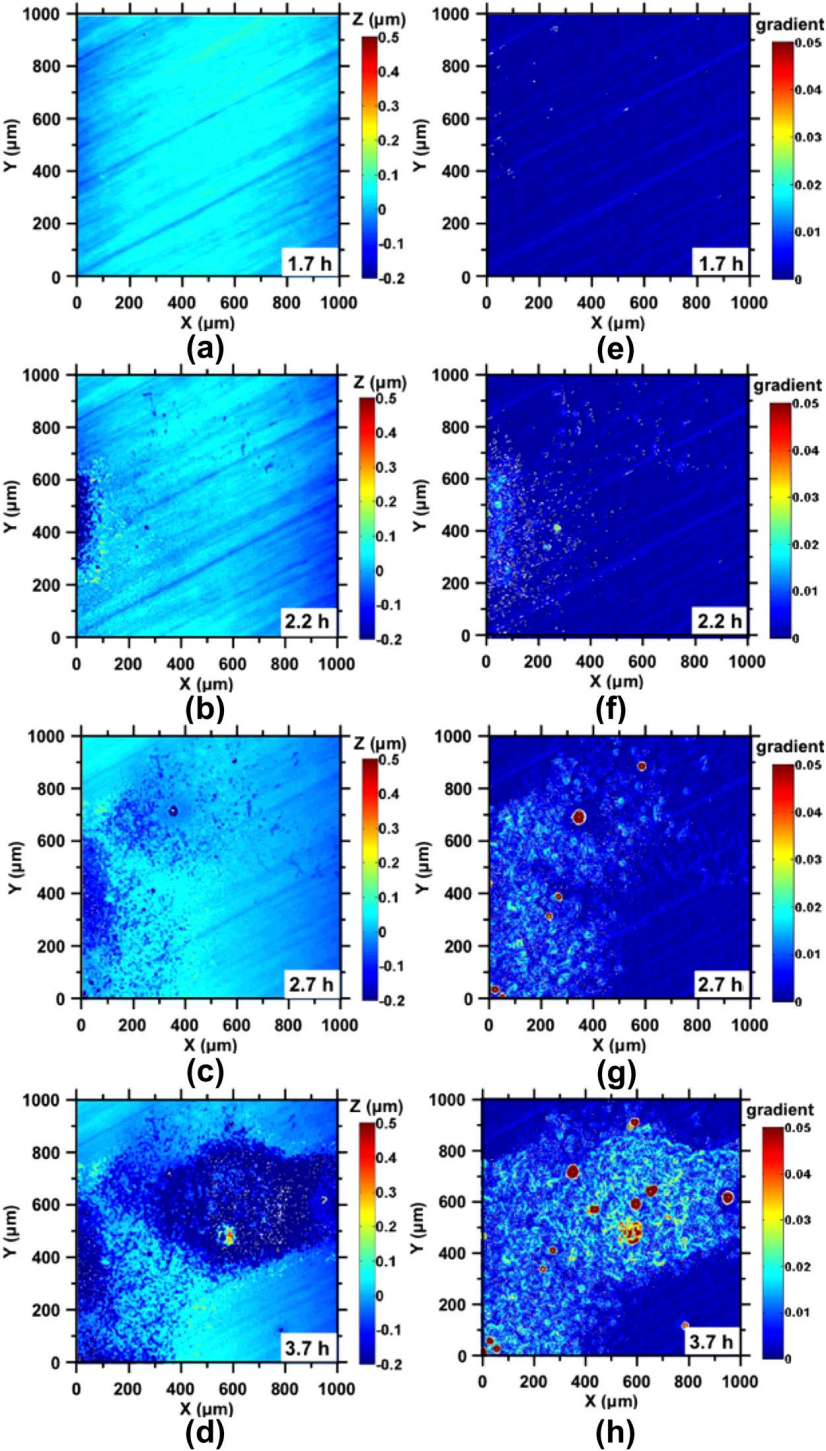
Representative illustrations of the height evolution of the steel surface following exposure to DI water after: **(a)** 1.7 h, **(b)** 2.2 h, **(c)** 2.7 h, and **(d)** 3.7 h. The corresponding gradient maps are shown in: **(e)** to **(h)**, respectively. Pitting sites were not evident until t = 1.7 h, as shown in **(a)** and **(e)**. After this time, pits nucleate as shown in **(b**,**f)**, and then continue to grow with concurrent surface roughening **(c**,**d**,**g**,**h)**. New pits formed around initially stabilized pits that are often isolated as evident in **(c)** and **(g)**.

**Figure 3:**
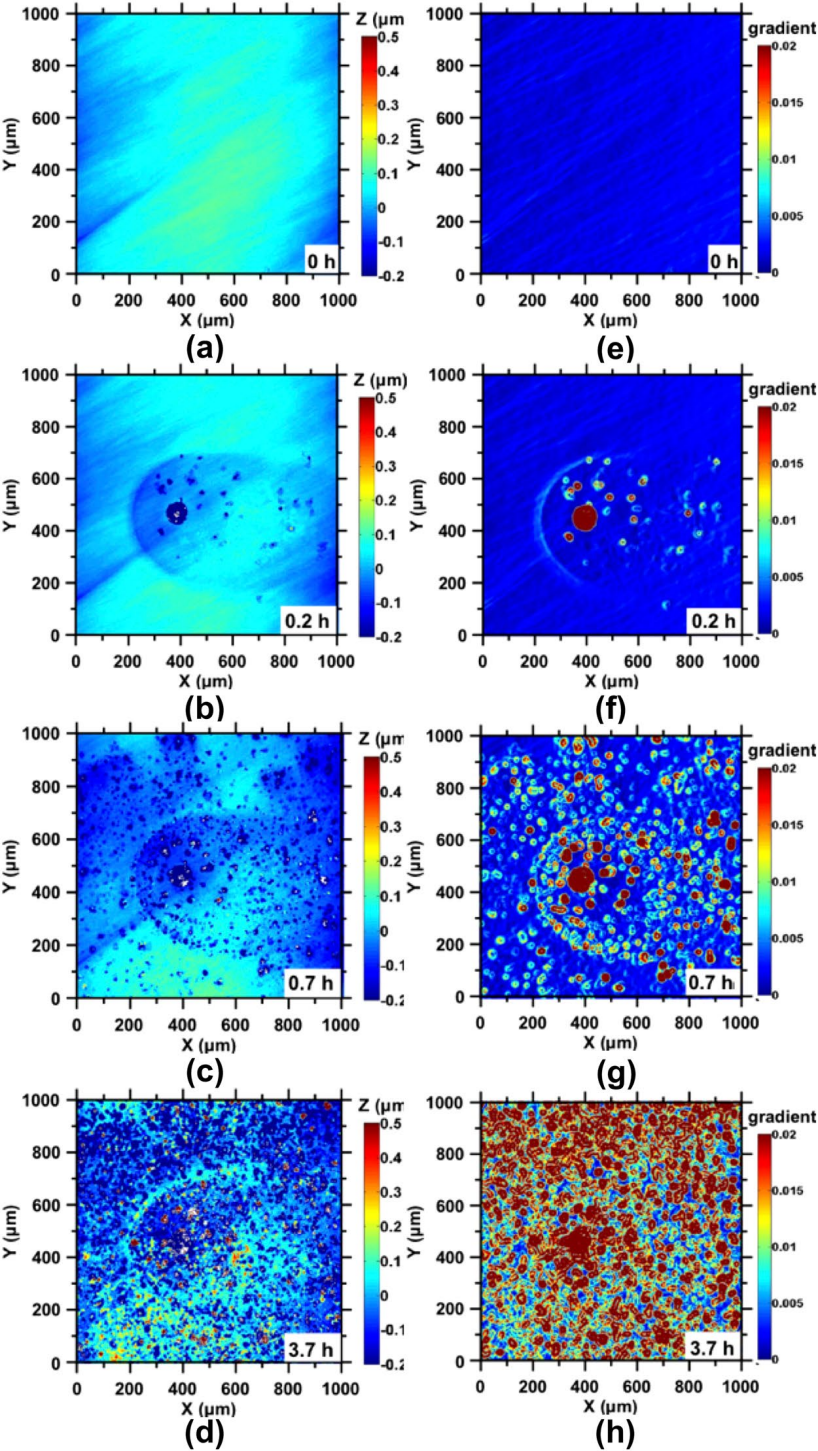
Representative illustrations of the height evolution of the steel surface following exposure to 100 mM NaCl for: **(a)** 0 h, **(b)** 0.2 h, **(c)** 0.7 h, and **(d)** 3.7 h. The corresponding gradient maps are shown in **(e)** to **(h)**, respectively. The end of the “dormant period” is marked by the development of circular basins around an initially formed isolated pit **(b**,**f)**. Significant pit nucleation and growth is observed within these basins initially, after which pit formation proceeds near-uniformly across the entire surface **(d**,**h)**.

The highly localized nature of corrosion reactions is highlighted in Figs 2–3 that represent large fields of view of 1000 µm × 1000 µm. For clarity, surface topography data are presented alongside its corresponding gradient map, which aids in the identification of high-slope features such as pitting sites. In general, in both DI water and 100 mM NaCl solution, pits nucleated as the surface roughened. While pit formation and surface roughening progressed in a similar manner in both DI water and NaCl solutions, expectedly, the progress of reactions is highly accelerated in the latter case. In DI water, an initial period of up to around 1.7 h is characterized by the absence of significant changes in surface morphology as indicated by Fig. 2(a). After this point, pit nucleation is accompanied by substantial surface roughening in the vicinity of pitting sites (e.g., see Fig. 2b,c,f,g). This initial nucleation period represents the time required to establish a critical local acidic pH that induces pitting^30^. Such pit nucleation is provoked by the passive dissolution current that triggers local instabilities in the alloy’s surface microstructure^30^. Significantly, as corrosion progresses, new pits form preferentially within the initially roughened/destabilized area (e.g., see Fig. 2d,h) which resemble a “circular basin”; due to localized acidification resulting from oxygen consumption within pits in these zones^31,32^. The formation of these circular basins, which have depths of 100-to-200 nm with respect to the uncorroded surface, is postulated to be on account of an (effective) *1/r*^2^ scaling in the corrosion current density that radiates from the geometric center of the circular basin(s) to the un-roughened (uncorroded) locations on the steel surface. These processes of pit nucleation, roughening and basin formation around pitting sites, and pit growth and proliferation, in time, resulted in a steel surface that exhibited evident discoloration characteristic of ‘rusting’.

Corrosion – while accelerated, follows similar dynamics – in steel exposed to 100 mM NaCl solution, as shown in Fig. 3. Expectedly, corrosion proceeds much faster in the presence of aqueous Cl^−^ with a considerable shortening in the time to pit nucleation, basin formation, and pit (and basin) growth (Fig. S2). For example, within 0.2 hours of exposure, distinct circular basins with depths on the order of 100-to-200 nm are observed around the initially formed isolated pits (see Fig. 3b), similar to those formed in DI water as described above. New pits nucleate within these basins (Fig. 3b,f), and in addition, pitting is observed outside the basin regions after longer periods of Cl^−^ exposure (Fig. 3c,g). This nature of extensive surface pitting was not observed in DI water even after >10 hours of exposure.

### Direct observation of the different stages in pit nucleation and growth

**Figure 4:**
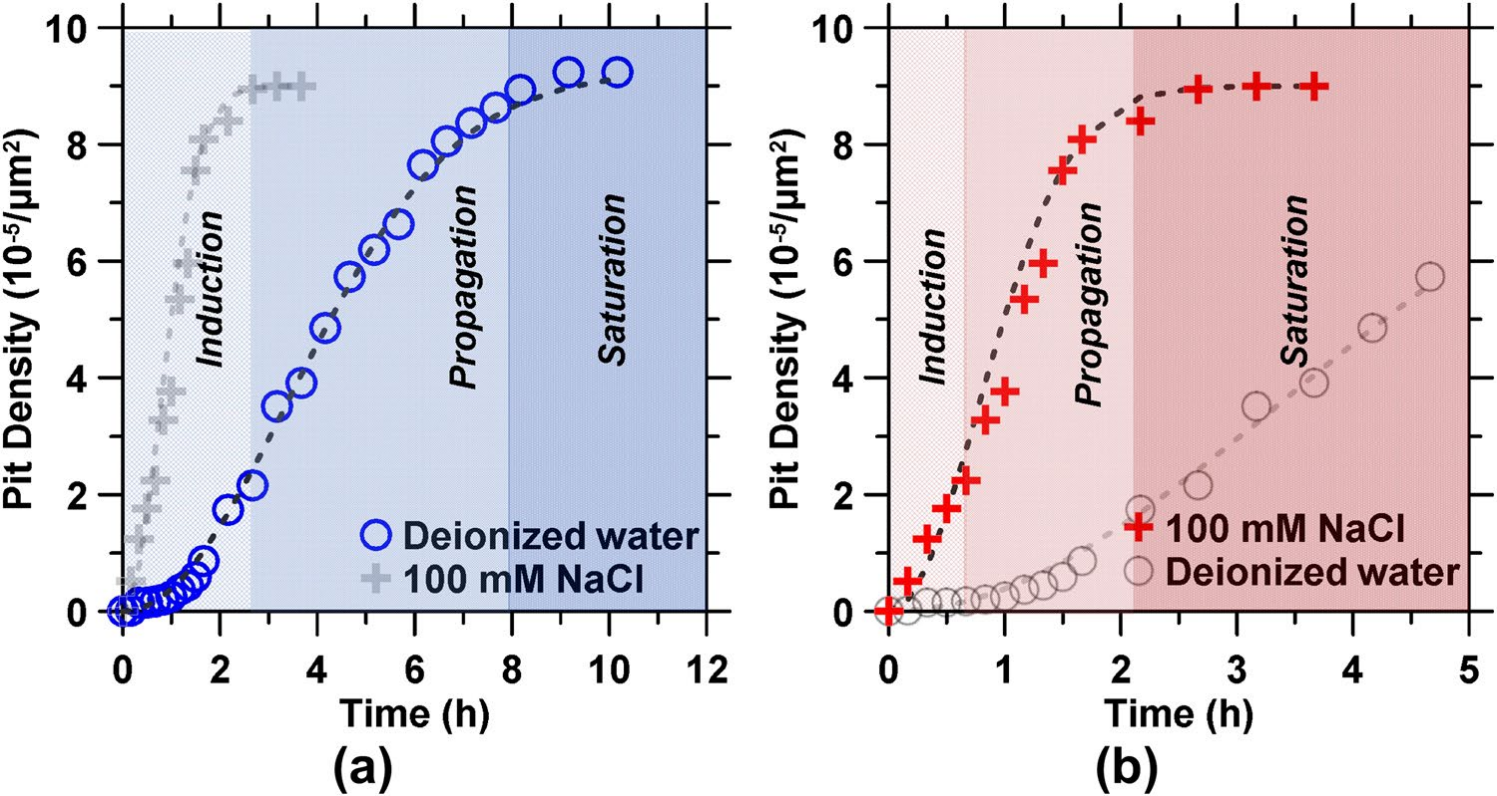
The pit density for steel surfaces reacting with: **(a)** DI water, and **(b)** 100 mM NaCl solution as a function of time. The respective best-fit curves obtained by fitting a modified second-order Avrami equation are shown by dashed lines. The shaded regions indicate the periods of induction, propagation, and saturation of pitting corrosion.

**Figure 5:**
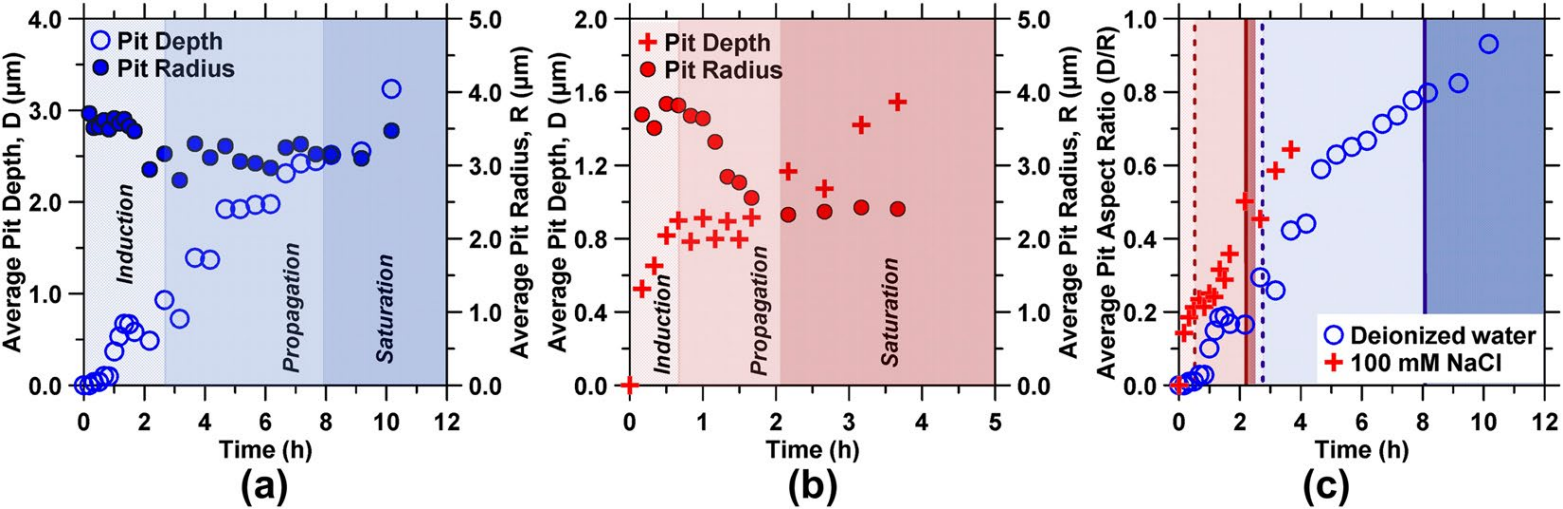
The evolution of the pit geometry following exposure to: **(a)** DI water and **(b)** 100 mM NaCl solution. The average depths, D (open circles in **(a)**, red crosses in **(b)**) and radii, R (closed circles in **(a)** and **(b)**) are plotted on the primary and secondary *y*-axis, respectively. **(c)** The average pit aspect ratios (i.e., AR = pit depth/pit radius, or D/R, unitless) are also given for DI water and 100 mM NaCl solutions. The shaded regions indicate the periods of induction, propagation, and saturation. The dashed and solid lines in **(c)** indicate the end of the induction and propagation periods, respectively, in both DI water and 100 mM NaCl.

The evolution of pitting was assessed by quantifying pitting parameters including the: (i) pit density (i.e., the total number of pits per unit area) which includes identification of transient pits, those that may have been obscured due to deposition of corrosion products within/over them, and newly formed and growing pits (Fig. 4), (ii) average depth and radius of pits present in the FOV (Fig. 5a,b), and, (iii) the average pit aspect ratio (i.e., the ratio of the pit depth to its effective circular radial opening, Fig. 5c).

Analysis of the pit parameters reveals three distinct stages of corrosion: (1) an *induction* period when pits nucleate but the surface undergoes negligible topographical changes, (2) a *propagation* period that is characterized by a rapid increase in the pit density, and (3) a *saturation* period when the rate of pit formation shows an asymptotic plateau (see Fig. 4)^8,33,34^. The time evolution of the pit density data which suggests the susceptibility for pit nucleation and corrosion rates^35^, can be described by a generalized exponential growth function which represents a two-dimensional (2D) nucleation and growth process^36^, as given by: $N=b(1-{e}^{-a{t}^{2}})$, where *N* and *t* are the pit density and time, and *a* and *b* are fitting parameters, respectively (Table 1). The maximum rate of increase in the (areal) density of pitting sites, *r*_*max*_, can be written as:$\,{r}_{max}=\sqrt{\frac{2a}{e}}b$. It should be noted that this functional form successfully describes the sigmoidal behavior of pit density evolution over time, and is not an attribution of specific mechanistic features. The times denoting the end of the induction and propagation periods, *t*_*ind*_ and *t*_*prop*_, respectively, are obtained by determining the saddle points from the third derivative of the 2D-Avrami equation (see Fig. 4 and Table 1).

**Table 1:** The best-fit parameters of the modified second-order Avrami equation that was fitted to the pit density data shown in Fig. 4.

	Parameters					
	a^*^ (/h^2^)	b^**^ (−)	R^2^	r_max_^§^(/h)	t_ind_^†^ (h)	t_prop_^‡^ (h)
Deionized water	0.043	923.32	1.00	164.24	2.53	7.96
100 mM NaCl	0.670	899.60	0.99	631.65	0.64	2.02

^*,**^Fitting parameters.

^§^Maximum rate of increase in the pit density.

^†^Time at the end of induction period.

^‡^Time at the end of propagation period.

The periods of induction, propagation, and saturation determined from the pit density evolution also delineate the evolution of the pit geometry as shown in Fig. 5. For instance, the beginning of the induction period in DI water is characterized by a stable average pit depth and radius, and thus, aspect ratio (Fig. 5a,c). Later, during the propagation and saturation periods, the pit depth increased continuously, while the pit radius remains near constant – suggesting deepening of existing pits, without the nucleation of any new ones. A marked contrast with the 100 mM NaCl solution was observed. Therein, the average pit depth increased steadily in both induction and saturation periods (Fig. 5b). On the other hand, the average pit radius transitioned from being initially stable (induction), to decreasing progressively during propagation due to the continual emergence of new and smaller pits (e.g., see Fig. 5b) achieving a stable value only in the saturation regime. Indeed, the propagation period is characterized by the rapid nucleation of small and shallow pits, resulting in a near constant average pit depth despite the deepening of existing pits – although this may also indicate the partial infilling of existing pits due to the deposition of corrosion products within them. In addition to the pitting parameters, the root mean square (RMS) surface roughness of the non-pitted regions also increased with time (see Fig. S3). Thus, in contrast to the pit density (see Fig. 4), a final saturation period was not observed for either pit depths or surface roughness indicating the persistence of corrosion reactions as shown in Fig. 5 and Fig. S3.

### Topographical manifestations of electrochemically induced pitting corrosion

Measurements over large FOVs at nanoscale height resolution enabled the monitoring and statistical analysis of the corrosion process. From the discussion above, it is clear that the corrosion of AISI 1045 steel is highly heterogeneous (Figs 2 and 3), and proceeds in multiple steps. Although the 1045 steel is expected to feature no passivation behavior when immersed in either DI water or 100 mM NaCl solutions (pH 5.8) based on Pourbaix diagrams (i.e., for the present conditions the steel simply bears an oxidation film; albeit one that does not feature passivation to corrosion)^37,38^, pit nucleation and the associated increase in surface roughness evolve progressively during the initial period of immersion; i.e., during the induction period when the rate of topography (and hence reaction) evolution is vanishingly small.

The induction period is thought to result from the presence of an oxidation film that forms following the exposure of steel to air, and that is presumed to be composed of iron oxides having a thickness of ≤20 nm^2,39,40^. It is important to highlight that induction pits not only contribute to the breakdown of this oxide layer but also catalyze corrosion in their vicinity, leading to the formation of the roughened shallow basins that feature an RMS roughness that is around 10× higher than their uncorroded surroundings (Figs 2c and 3b). On account of their substantial depth (>100 nm), these basins are expected to be fully activated since the surface retreat in these zones is much larger than the typical thickness of the oxide layers (i.e., passivated or not) that form on steel surfaces up on exposure to air, or caustic solutions^41–43^. As such, the formation of these basins clearly marks an increase in localized corrosion activity that results from acidification caused by oxygen consumption during pitting corrosion. The rapid consumption of oxygen within the pits and its comparatively slow replenishment via diffusion results in localized potential and pH differences, i.e., from the bottom of a pit to the perimeter of the pit opening^44,45^, leading to sustained vertical propagation of corrosion pits, as well as formation of basins due to lateral expansion of the pit opening. The inadvertent hydrolysis of ferric ions and the formation of hydroxy-oxides (Fig. S4) are expected to result in further acidification and expansion of the pit mouth^46,47^. In addition, a recently identified electric field concentration near the opening of the pit also facilitates the formation of a circular basin – due to electrically stimulated dissolution in the vicinity of the pit mouth^20^. In contrast, the area outside these basins exhibits limited pitting, particularly in the case of DI water, and at small exposure times, even in the presence of Cl^−^ ions.

Cl^−^ ions significantly accelerate steel corrosion^6,7,48^, as manifested by the following: (a) an enhanced and accelerated tendency for roughened basins to develop, (b) nucleation of pits within, as well as outside the basin regions at longer exposure times, and, (c) an aggressive autocatalytic pitting corrosion. These observations can be confirmed from analysis of the pit density curves shown in Fig. 4 (Table 1). Based on the results of fitting the exponential growth function, it was found that the induction period is much less evident and the pit density propagation rate is around 4× faster in the presence of 100 mM NaCl, as compared to DI water. Numerous studies have suggested that pitting of oxidation/passivation film-bearing metals in the presence of Cl^−^ occurs via mechanisms including film penetration^49^ and (Cl^−^) ion-adsorption^1,6^. As such, the localized oxidative dissolution of either the oxide film or the underlying steel, which results in the formation of the pits and basin regions is accelerated when Cl^−^ ions are present^7^. First, the heightened adsorption of Cl^−^ in localized domains is expected to cause local dissolution and thinning by producing ionic point defects and regions of high local current density. This is consistent with the shortened induction period that is observed when Cl^−^ ions are introduced as compared to DI water. On the other hand, the penetration of Cl^−^ into the air-formed oxide film would result in a contaminated film which has a higher ionic conductivity than the pristine film. The observation of pit nucleation outside the locally activated basin regions supports the hypothesis that the penetration of Cl^−^ ions is a relevant, but secondary mechanism by which pitting occurs. This is based on the observation that, initially, the pit density outside the basins is far lower than within the basin regions. It should be noted that uniformly distributed pitting occurred only at later times, consistent with an initial period of minimal corrosion in these regions. This implies a “characteristic time” that is associated with the penetration into the air-formed oxide layer of Cl^−^ species to a critical depth, as a function of the local solution and solid (protective film) thickness and composition.

**Figure 6:**
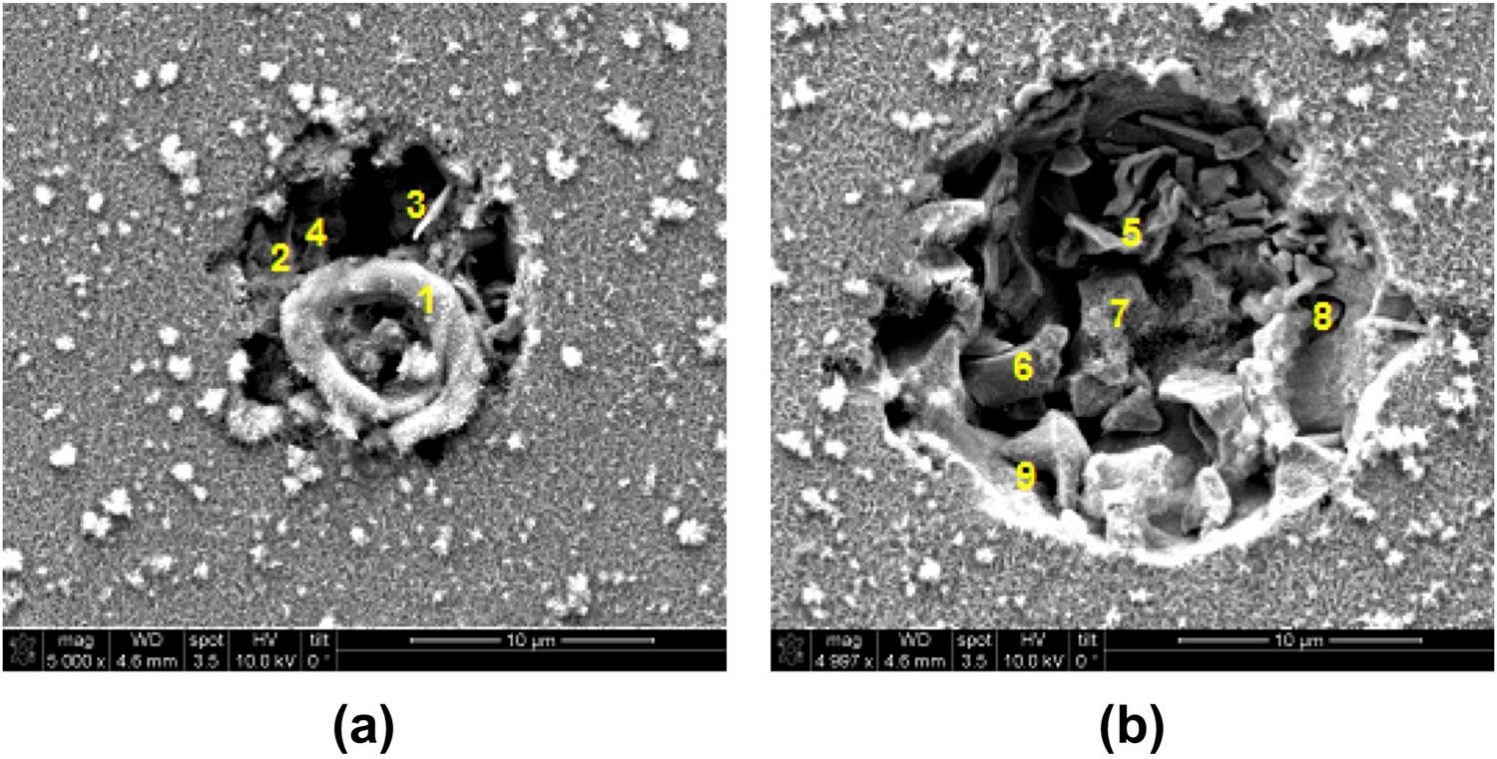
SEM micrographs of a representative induction pit on AISI 1045 steel reacting with 100 mM NaCl for: **(a)** 0.1 h, and **(b)** 3.3 h, showing radial expansion with time. In **(a)**, sample areas labeled 1 to 4 have the following Mn and S contents: 0.1%, 2.3%, 22.1%, and 17.3%, and 0.1%, 0.2%, 0.1%, and 0%, respectively, as measured by SEM-EDS (in mass %). At longer reaction times, in **(b)**, sample areas labeled 5 to 9 have the following Mn and S contents: 2.2%, 5.0%, 2.8%, 2.8%, and 1.3%, and 0.1%, 0.1%, 0.01%, 0.2%, and 0.0%, respectively (in mass %). The relatively high concentrations of Mn and S are consistent with the presence of a MnS inclusion at this pitting site.

The origin of the induction pits merits further consideration. For example, induction pits are expected to arise at defect sites, e.g., in AISI 1045 steel, at interfaces between ferrite and cementite or at locations of embedded secondary-phase particles, which are weakly passivated or thermodynamically unstable (i.e., vis-à-vis the bulk alloy)^1,7,8^, and therefore can serve as nucleation sites for pit formation. Indeed, our SEM-EDS analysis reveals that induction pits formed on AISI 1045 steel surfaces typically feature high concentrations of sulfur and manganese, whose abundances generally decrease as the pits grow (e.g., see Fig. 6). This observation supports the idea that pits may originate – although not exclusively – from the dissolution of manganese sulfide (MnS) inclusions which form in steel during its processing^22,50^. These inclusions, which display a high chemical affinity for oxygen and water, can rapidly solubilize, leaving behind pits that can grow to become greater than 20 µm in diameter^51^. More problematically, halide/Cl^−^ ions are also known to preferentially adsorb at such inclusion sites^52^.

Beyond the induction period, pitting dramatically accelerates. It should also be noted that the propagation pits tend to continuously deepen (grow) despite the saturation in pit density (e.g., see Fig. 5). In water, the anodic corrosion reaction is given by: $Fe\to F{e}^{2+}+2{e}^{-}$, which generally occurs within the pits, whereas the initial cathodic reactions can be written as: $\frac{1}{2}{O}_{2}+\,{H}_{2}O+2{e}^{-}\to 2(O{H}^{-})$, which is limited by oxygen diffusion. Once the steel starts to corrode rapidly, the rate of oxygen consumption within the pit exceeds its replenishment through diffusion from the region outside the pit, leading to the local accumulation of the metal cations, and hydrolysis through the reactions: $F{e}^{2+}+{H}_{2}O\to FeO{H}^{+}+{H}^{+}$, and $F{e}^{2+}+2{H}_{2}O\to Fe{(OH)}_{2}+2{H}^{+}$. Local acidification in the microenvironment arises from the release of protons due to chemical, electrochemical, and geometrical gradients within and around the pit sites^49^. The formation of the basin regions and the emergence of different stages of pitting emanate from the kinetics of this acidification process. In general, the microenvironment within the pit is characterized by low pH and high ionic concentrations, particularly of Cl^−^ (when present), which may also facilitate the formation of salt films^53^ within the pits^8,54–58^. As shown in Figs 4 and 5, the deepening of pits proceeds even after saturation in pit density is achieved, indicating a highly aggressive microenvironment of the near-surface region which leads to the recess of the whole surface. This is consistent with the surface height change profiles provided in Fig. 1, showing the continued build-up of corrosion products during the saturation stage.

## Conclusions

Taken together, direct observations of pitting corrosion – from the nano- to the micro- scales – in DI-water and Cl^−^containing solutions highlight that Cl^−^ ions enhance corrosion by promoting the breakdown of air-formed oxide films that are expected to be present on steel surfaces, and by assisting in maintaining aggressive pitting microenvironments. It is clarified that pitting is the dominant form of corrosion in AISI 1045 carbon steel and is fundamental to the onset and progression of such degradation. It should be noted that although the present study investigates the behavior of corrosion of a non-passivated surface, the observations can be related to passivated surfaces experiencing localized transpassive conditions. For instance, it may be implied that similar surface evolutions may occur in carbon steels undergoing crevice corrosion or during galvanic coupling with a cathodic material although the bulk steel is itself in a passivated condition. Importantly, the measured statistical surface regression (reaction) rates are valuable for estimating longer-term corrosion rates as may be obtained from electrochemical analysis such as Tafel polarization. An important outcome of this work is that it offers a quantitative, direct and unambiguous basis to correlate surface morphology evolutions of reacting surfaces with known electrochemical processes such as corrosion, by carefully examining the time-dependent evolution of such deleterious reactions.

## Methods

### Materials and sample preparation

**Figure 7:**
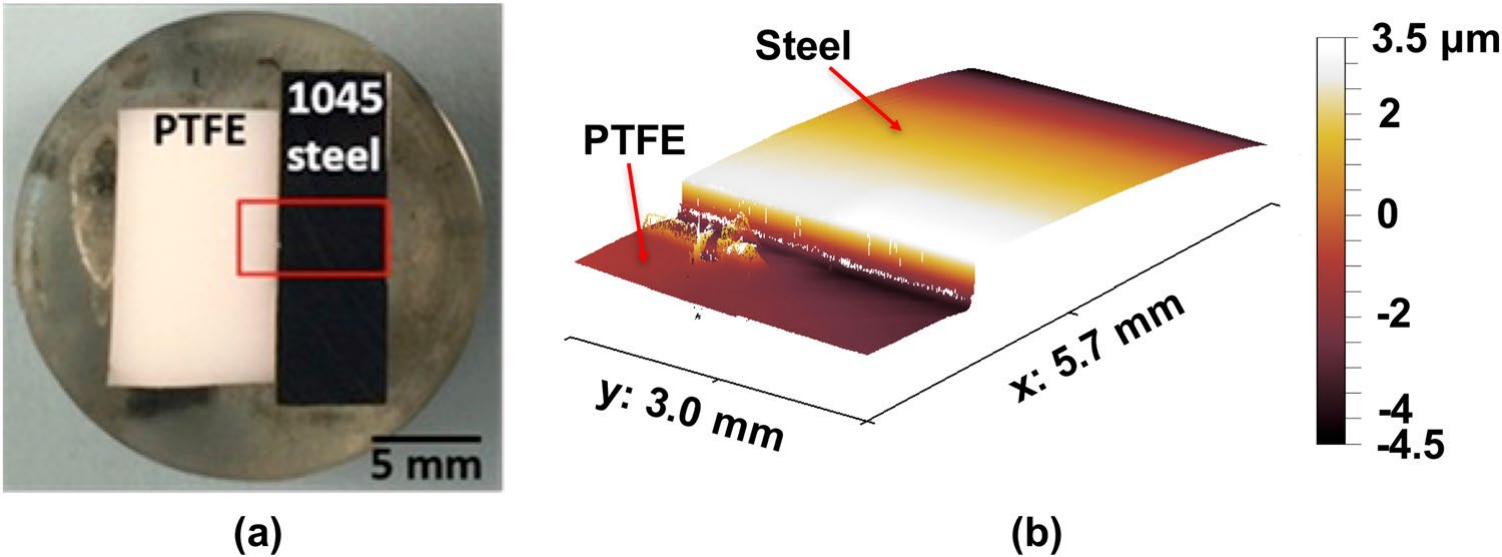
(**a**) An image of the plane-polished epoxy “puck” in which the PTFE and AISI 1045 steel were embedded, and, **(b)** A representative VSI image of the reference (PTFE) and steel samples that are embedded beside each other.

Commercially available AISI 1045 steel was sectioned into cuboid specimens with dimensions of 1.7 cm × 0.8 cm × 1.7 cm (length × width × height) using a low-speed precision saw fitted with a diamond wafering blade (Buehler, IsoMet 1000). This medium-carbon steel features a nominal composition in mass % of: C (0.4–0.5%), P (0–0.04%), Mn (0.6–0.9%), S (0–0.05%), Si (0.2–0.3%), and Fe (98.2–98.9%). Polytetrafluoroethylene (PTFE) which is well-known to be inert in corrosive solutions^59–61^, was used as the reference material for VSI imaging as noted below. In addition, PTFE is hydrophobic – therefore it resists the deposition of corrosion products on its surfaces. Coupons of PTFE were sectioned and embedded adjacent to the steel sample in an epoxy ‘puck’ (see Fig. 7a) to facilitate handling. After curing the resin for at least 10 hours at ambient temperature, the hardened puck was progressively polished using 400-, 600-, 800-, and 1,200-grit sandpaper and 6-, 3-, 1-, ¼-µm diamond pastes. The polished sample was cleaned by ultrasonication in ethanol for 2 minutes, and then dried in a stream of ultrahigh purity (UHP) nitrogen gas.

### Vertical scanning interferometry (VSI)

The evolution of the steel surface’s morphology during corrosion was monitored using a vertical scanning interferometer (NewView 8200, Zygo Corporation). To enable imaging of large corroding areas, a 5× Mirau objective (numerical aperture, N.A. = 0.13) was used which offers a lateral resolution (i.e., pixel size) of 1.63 µm in the *x* and *y* directions. The nominal vertical resolution is on the order of 2 nm. With this configuration, the field of view of each VSI image is 1.7 mm × 1.7 mm and can be expanded by merging (“tiling”) multiple images that are imaged along a grid (e.g., a grid comprising A × B images consists of A.B images that are taken sequentially). Tiled areas consisting of the steel and reference surfaces, and having nominal dimensions on the order of 5.7 mm × 3.0 mm, were examined (see Fig. 7b). The observable surface area of the steel is around 10.7 mm^2^, and consists of 4.03 × 10^6^ pixels. This large area allows for the observation of hundreds of corrosion sites simultaneously.

To initiate corrosion, the polished sample was immersed in 70 mL of either: (*i*) 18 MΩ·cm deionized (DI) water, or, (*ii*) 100 mM NaCl solution, which was prepared by adding ACS reagent grade NaCl to 18 MΩ·cm DI water. The measured pH of DI water and the Cl^−^-containing solution was around 5.6–5.8, and the reaction temperature was 25 ± 3 °C. The corrosive solution (70 mL) contacted a surface with a nominal area of 17 mm × 8 mm resulting in a surface (solid)-to-volume (liquid) ratio of 0.002 mm^−1^ (S/V)^62^. The sample was periodically removed from the solution and dried with a nitrogen gas stream prior to surface topography characterization. Corrosion activity was assessed periodically for up to 10 hours in DI water, and 3.5 hours in 100 mM NaCl solution, at which time the steel surface was almost completely covered by (visible) corrosion products (“rust”).

Surface topography data (i.e., in the form of three-dimensional (3D) images) acquired by VSI were processed and analyzed using MATLAB©. The analysis was carried out using bespoke scripts that performed the following operations: (1) 3D images of a given (imaging) area that were acquired at different reaction times were aligned; (2) corrosion sites were identified; and (3) the geometry (i.e., radius, depth, and aspect ratio) of the sites was characterized, all as a function of contact time with the reaction solution. If a given pixel location did not contain height data, such data was retrieved by interpolation from a 4^th^ order function fitted to the known adjacent (local topography) data. To align the 3D images, the PTFE reference was marked with a sharp tool (Fig. S4). Then, an area of an arbitrary size enclosing the *mark* was cropped from the 3D image and then converted into a 2D grayscale image. This 2D image was then aligned with that collected at *t* = 0 using an image-registration algorithm that only permitted translation and rigid rotation within the *x-y* plane. The image-registration algorithm produced a 2D transformation matrix, which was then applied to the entire 3D image. Aligning the 3D surface topography using the reference mark enabled precise identification of transient and newly formed features on the steel surface as corrosion evolved. In addition to lateral alignment, the 3D images were also aligned vertically (in the *z*-coordinate) by matching the average heights of equivalent areas on the PTFE reference at *t* = 0 and at later times (see Fig. S4).

#### Strategy for pitting site identification

**Figure 8:**
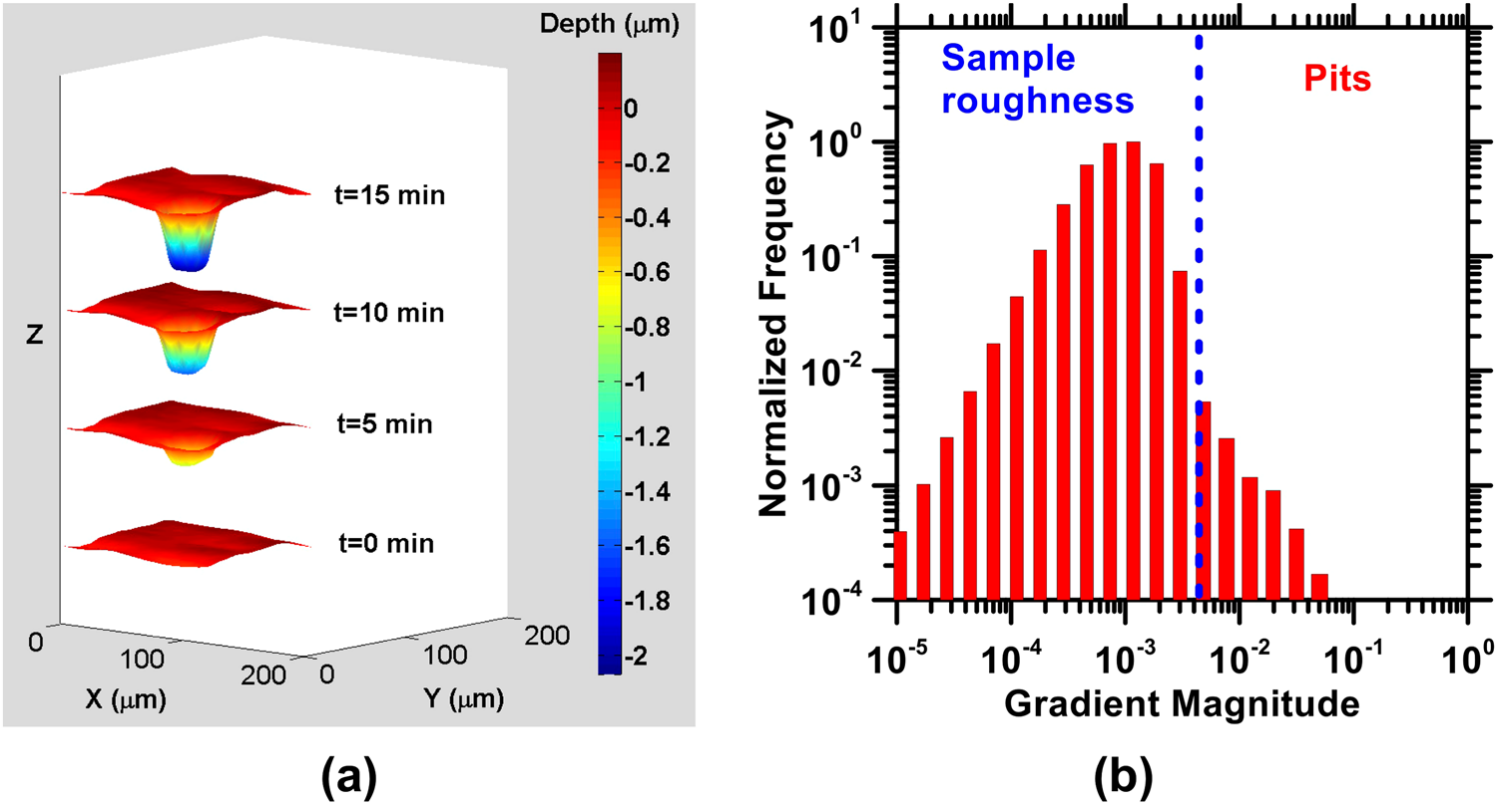
(**a**) An illustration of pit growth on AISI 1045 steel in 100 mM NaCl. From the bottom to the top, the diagram shows depth evolution after 0 min, 5 min, 10 min, and 15 min of solution exposure. After 15 min, the pit depth was around 2 μm, and **(b)** The methodology for identifying pitting sites on the sample surface. In general, pits are differentiated from the sample’s roughness using a threshold gradient value shown by the dashed blue line.

Although general topographical features could be readily identified from the unprocessed 3D images (e.g., the average height of the steel surface relative to that of the inert reference), identifying transient and fine-scale features such as pitting sites requires additional effort. Figure 8(a) shows a surface corrosion site profiled by VSI that was identified as a pit. Such pit identification was accomplished in two steps. First, each pixel located on a 3D image was classified as either a part of a pit or not, based on the magnitude of its gradient (i.e., slope in 2-dimensions, $\sqrt{{(\frac{\partial z}{\partial x})}^{2}+{(\frac{\partial z}{\partial y})}^{2}}$, where *z* is the height of each pixel) compared to a threshold value. The threshold for gradient-based identification of pits was selected based on the distribution of gradients in each image (e.g., see Fig. 8b). A typical value of such a threshold is 1%, i.e., if the maximum gradient frequency distribution in a VSI image is *F*, then pixels with a gradient magnitude below that at 0.01 *F* are considered to be non-pitting sites, whereas pixels with a gradient magnitude greater than that at 0.01 *F* are identified as pits (Fig. 8b).

It is recognized that pits could also be identified based on an absolute height change with respect to the initial surface. However, such methods are ineffective in distinguishing localized height decreases (i.e., pit formation) from uniform surface retreat (i.e., uniform dissolution). In contrast, the use of a local gradient-based method allows for pits to be identified based solely on their geometrical characteristics, and the gradient of their proximate surroundings. However, since the gradient method exploits the idea that the walls of the pit are expected to be high-slope features, it tends to ignore the region at the bottom of a pit, which often has a low gradient. Thus, to ensure that a pit was identified in its entirety, a convex hull algorithm was implemented (Fig. S5). First, all neighboring pixels initially identified as belonging to pits (e.g., pixels at the walls of the pits) were grouped^63^. Then, a 2D convex hull was drawn around each group of pixels, based on their *x* and *y* coordinates, to indicate an area that is a potential pitting site. Based on overlaps in areas belonging to different potential pitting sites, the grouping of points was iteratively updated. A new convex hull was then drawn around points belonging to two overlapping areas, hence consolidating the areas into one pit. This process was performed iteratively, until the number of pits converged to a stable value. Each pit was assigned a unique identifier for further analyses.

#### Characterizing pit geometry, transience, and surface evolutions

Once pitting sites were identified, analyses were carried out to characterize pit nucleation and growth. To establish the baseline surface used to correct for tilt and curvature, first, a 4^th^ order polynomial was fitted to the 3D image excluding all identified pits, to capture the surface curvature (Fig. S6). The roughness of the steel surface without the pits, ${R}_{rms}$, is calculated with respect to this baseline surface as:$\,{R}_{rms}=\sqrt{\frac{{\sum }_{i=1}^{n}d{z}^{2}}{n}}$, where *n* is the number of data points, and *dz* is the distance to the baseline surface. The depth of each pit was defined as the distance between the lowest point in the pit and the baseline surface (Fig. S6). On the other hand, the pit radius was calculated from the measured pit area, assuming that the pits can be circumscribed by a circle. By analyzing overlapping pits (i.e., the same pitting sites) in time-spaced surface topographies, the nucleation and growth process of each pit can be studied. This permits quantification of pit density as a function of time, and the evaluation of pit growth rates, while being able to distinguish existing from new pits at each time interval.

### Data availability

The analysis scripts and datasets generated during and/or analyzed over the course of the title study are available from the corresponding author up on reasonable request.

## Electronic supplementary material

Supporting Information
