# Supplementary material for: Direct observation of pitting corrosion evolutions on carbon steel surfaces at the nano-to-micro- scales
Source: Sci Rep. 2018 May 22;8:7990. doi: 10.1038/s41598-018-26340-5 (PMC5964123; doi:10.1038/s41598-018-26340-5)
Supplement: Supplementary file 1 — Supporting Information [file 41598_2018_26340_MOESM1_ESM.pdf]

Direct observation of pitting corrosion evolutions on carbon steel surfaces at the nano-to-micro- scales

Peng Guo (,†), Erika Callagon La Plante (*,†), Bu Wang (†), Xin Chen (†), Magdalena Balonis (‡,§), Mathieu Bauchy (††), and Gaurav Sant (†,‡,‡‡,**)*

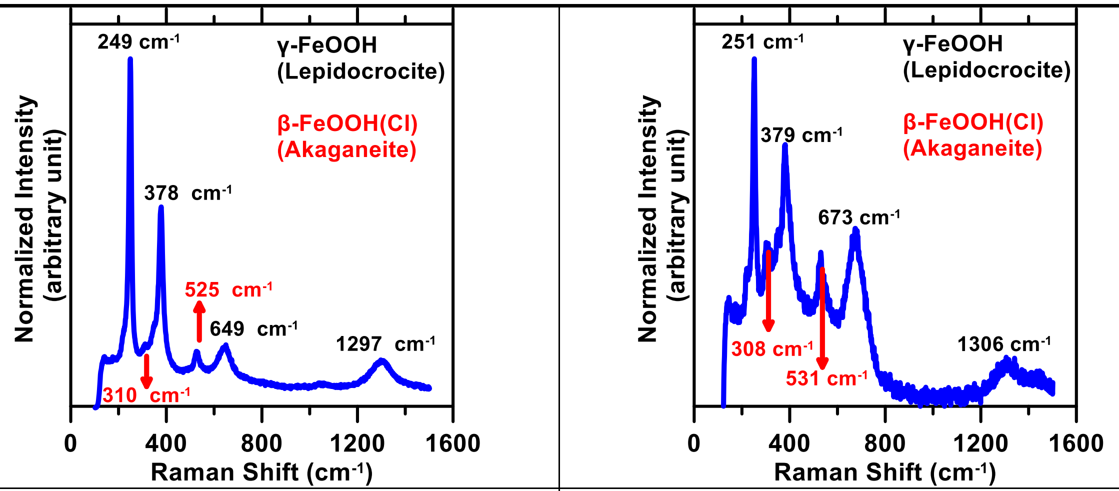

(a)	(b)

Figure S1: Raman spectra of AISI 1045 steel surfaces reacted for 24 hours with: **(a)** 10 mM NaCl, and **(b)** 100 mM NaCl solutions. In both cases, lepidocrocite (γ-FeOOH) (labeled with black text) and akaganeite (β-FeOOH(Cl)) (labeled with red text) are detected, consistent with previous observations.

[*]Both authors contributed equally to this work.
[†] Laboratory for the Chemistry of Construction Materials (LC^2), Department of Civil and Environmental Engineering, University of California, Los Angeles, CA 90095, United States
[‡] Department of Materials Science and Engineering, University of California, Los Angeles, CA 90095, United States
[§] Department of Bioengineering, University of California, Los Angeles, CA 90095, United States
[††] Laboratory for the Physics of Amorphous and Inorganic Solids (PARISlab), Department of Civil and Environmental Engineering, University of California, Los Angeles, CA 90095, United States
[‡‡] California Nanosystems Institute, University of California, Los Angeles, CA 90095, United States
[**] Corresponding Author: G. Sant, Email: gsant@ucla.edu, Phone: (310) 206-3084

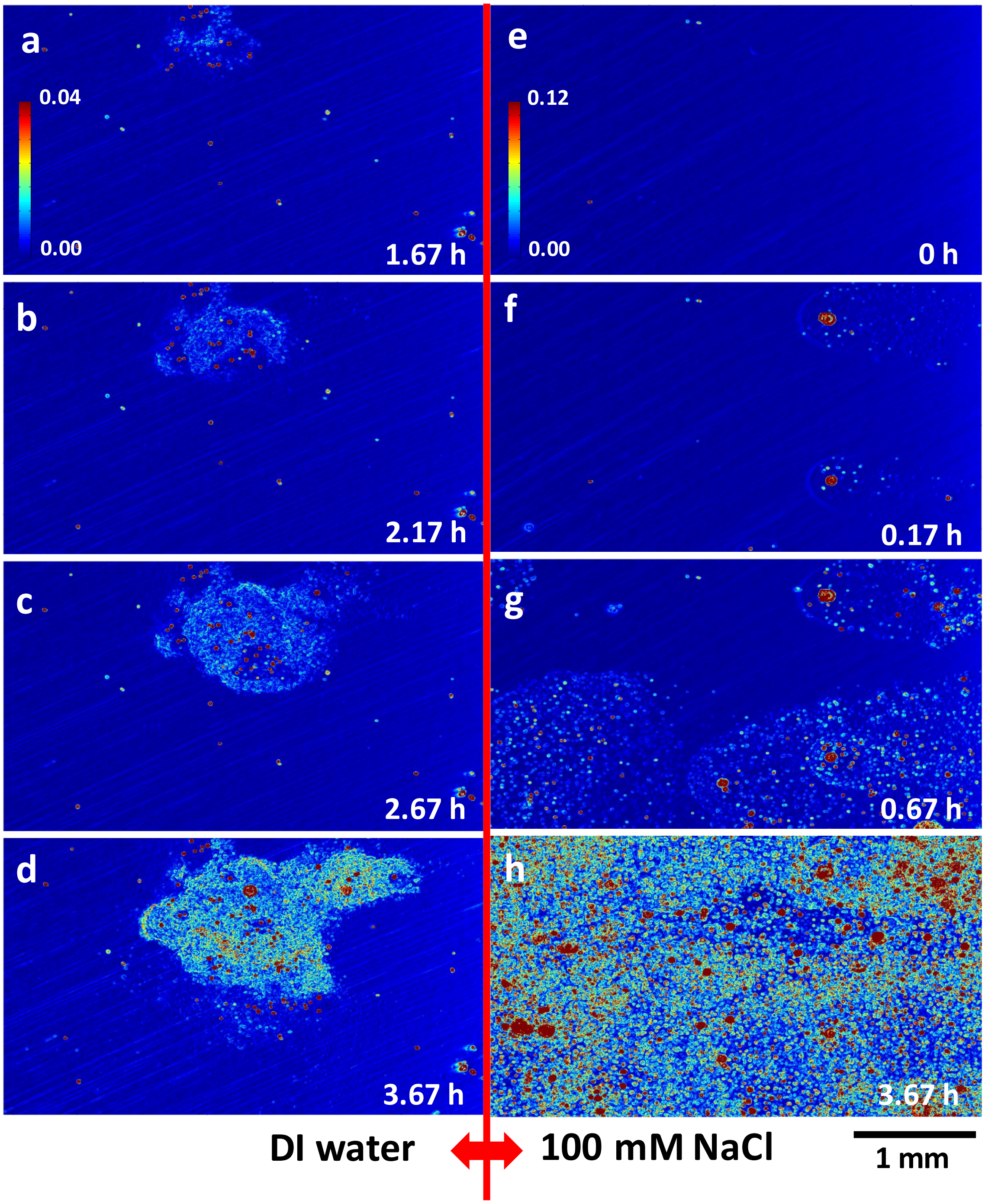

Figure S2: Gradient maps of corroding 1045 steel surfaces showing circular basins formed in **(a-d)** deionized water, and **(e-h)** 100 mM NaCl solution. These roughened circular basins are depassivated regions in which pits preferentially nucleate. As corrosion proceeded, the basins expand and eventually coalesce covering the entire FOV, and in time, the sample surface.

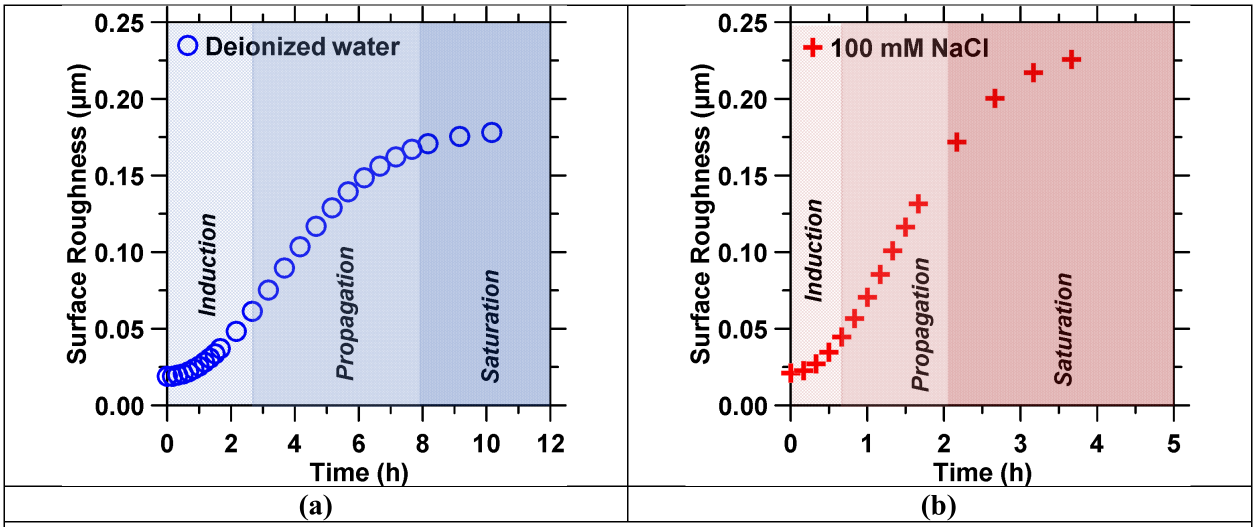

(a)	(b)

Figure S3: The RMS surface roughness evolution in non-pitting zones as a function of time in AISI 1045 steel reacted with **(a)** DI water and **(b)** 100 mM NaCl solution. The shaded regions indicate the periods of induction, propagation, and saturation.

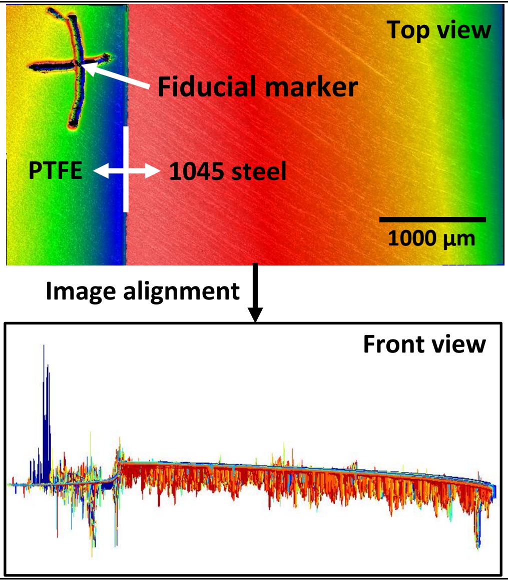

Figure S4: An illustration of the VSI 3D image alignment method. **(top)** A fiducial mark placed on the reference PTFE surface is used to align all VSI 3D images to the t_0 image. **(bottom)** An example wherein 50 (3D) images are aligned using this method.

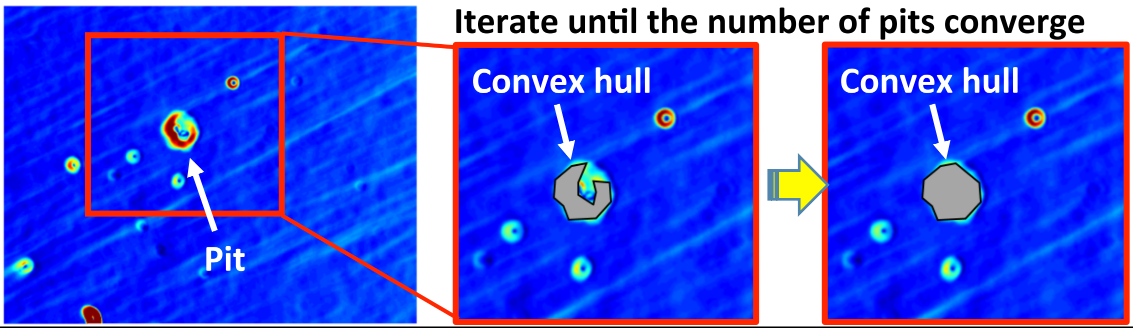

Figure S5: An illustration of the pit identification process using the convex hull method. **(left)** A gradient map of the steel surface showing a pit to be identified. **(middle)** A 2D convex hull is drawn on the target pit. Initially, the convex hull may not be able to completely identify a pit. **(right)** The convex hull algorithm is iterated until the number of pits converges to a stable value to ensure that the convex hull covers the specific pit completely.

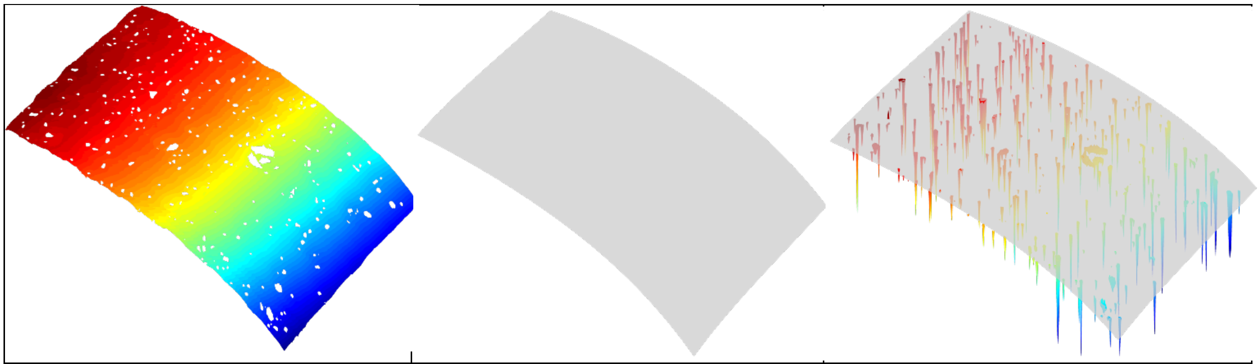

**(a)**	**(b)**	**(c)**

Figure S6: An illustration of the method employed to calculate pit depth. **(a)** A VSI image of the non-pitting area of the sample surface. Note that the pits have been removed from the data for ease in visualization. A fourth-order polynomial is fitted **(a)** to determine the baseline surface, **(b)**, from which pit depths are obtained, as shown in **(c)**.
